# Characterisation of Casein Kinase 1.1 in* Leishmania donovani* Using the CRISPR Cas9 Toolkit

**DOI:** 10.1155/2017/4635605

**Published:** 2017-11-29

**Authors:** Daniel Martel, Tom Beneke, Eva Gluenz, Gerald F. Späth, Najma Rachidi

**Affiliations:** ^1^Institut Pasteur and INSERM U1201, Unité de Parasitologie Moléculaire et Signalisation, Paris, France; ^2^Sir William Dunn School of Pathology, University of Oxford, Oxford, UK

## Abstract

The recent adaptation of CRISPR Cas9 genome editing to* Leishmania* spp. has opened a new era in deciphering* Leishmania* biology. The method was recently improved using a PCR-based CRISPR Cas9 approach, which eliminated the need for cloning. This new approach, which allows high-throughput gene deletion, was successfully validated in* L. mexicana* and* L. major*. In this study, we validated the toolkit in* Leishmania donovani* targeting the flagellar protein PF16, confirming that the tagged protein localizes to the flagellum and that null mutants lose their motility. We then used the technique to characterise CK1.1, a member of the casein kinase 1 family, which is involved in the regulation of many cellular processes. We showed that CK1.1 is a low-abundance protein present in promastigotes and in amastigotes. We demonstrated that CK1.1 is not essential for promastigote and axenic amastigote survival or for axenic amastigote differentiation, although it may have a role during stationary phase. Altogether, our data validate the use of PCR-based CRISPR Cas9 toolkit in* L. donovani*, which will be crucial for genetic modification of hamster-derived, disease-relevant parasites.

## 1. Introduction

The protozoan parasite* Leishmania* is the causative agent of leishmaniasis, which has several clinical forms depending on the species, including cutaneous (e.g.,* L. major* and* L. mexicana*), diffuse cutaneous, mucocutaneous, and fatal visceral leishmaniasis (e.g.,* L. donovani*) [1, 2].* Leishmania* goes through several extracellular developmental stages in the insect vector, from nonvirulent procyclic to virulent metacyclic promastigote forms [3], and one intracellular stage, the amastigote form, which resides inside the phagolysosome of the mammalian host macrophages. In recent years, omics systems-wide analyses, particularly RNA-Seq, have been applied for many purposes such as the determination of disease phenotype, the mode of action of drugs, or the identification of drug-resistance markers [4, 5]. These technologies have also dramatically improved our knowledge of* Leishmania* biology [4]. However, knowing the genes that are differentially regulated under different conditions is only the prelude to understand their role. This is particularly important for* Leishmania* as more than 50% of the genes encode hypothetical proteins [6]. One major bottleneck for their characterisation is the absence of a* Leishmania*-specific genetic toolbox that could overcome different parasite-specific limitations such as the absence of RNA-interference machinery in the subgenus* Leishmania*, a stark contrast to* Trypanosoma brucei*, where this technique greatly contributed to a better understanding of the biology of this parasite over the past decade [7, 8].

Although possible, genetic engineering has been particularly challenging and time-consuming in* Leishmania *parasites [9].* In locus* tagging of a gene of interest (GOI) requires multiple steps of cloning to assemble a cassette that could be integrated at the 5′ or the 3′ end of the gene [10]. Furthermore, the traditional gene targeting method involving homologous recombination requires the generation of a cassette containing an antibiotic selection marker gene flanked by 300 to 900 bp of both the 5′ and 3′UTR of the GOI to direct integration into the genome [10, 11]. This strategy has many drawbacks [12]: (i) for a diploid asexual organism such as* Leishmania*, at least two rounds of transfection are required [13], and (ii) heterozygous transfectants need to be selected before the second round can be performed. The generation of a knockout strain is thus time-consuming and can favor misintegration of the targeting cassette elsewhere in the genome or parasite compensatory adaptations if the deleted gene is important for survival. This is particularly true for* L. donovani*, as several studies have shown its ability to adapt to stressful conditions by copy number variations leading to gene amplification, gene deletion, or aneuploidy [14, 15]. This genome instability further complicates genetic engineering, as the presence of additional chromosomes requires additional rounds of transfection to obtain a complete deletion of the GOI. A simpler and more efficient method is therefore required to decipher* L. donovani *biology.

The clustered regularly interspaced short palindromic repeats (CRISPR)/CRISPR-associated protein 9 (Cas9) has been used, since 2013, as a genome editing tool for a large number of organisms including yeast and mammalian cells, and has subsequently been adapted for several unicellular human pathogens such as* Plasmodium falciparum* and* Trypanosoma cruzi *[16]. The recent adaptation of CRISPR Cas9 for* Leishmania *spp. has opened a new era for* Leishmania *genetic manipulation [17–19]. However these early methods required the cloning of at least the single guide RNA (sgRNA) in an expression vector, which increases the time necessary to generate a knockout and prevent the use of these methods to perform high-throughput gene tagging or deletions. In a recent paper, Beneke et al. described the development of a new PCR-based CRISPR Cas9 toolkit allowing rapid and precise gene modification, which was successfully applied to* L. mexicana*,* L. major*, and* Trypanosoma brucei *[20]. Parasites stably expressing hSpCas9 and T7 RNA polymerase were transfected with PCR fragments corresponding to the sgRNA and the donor DNA cassettes to generate knockout or tagged parasites in only one week. This method is perfectly suited to generate knockout parasites in a high-throughput fashion, as no cloning is required [20]. The toolkit includes simple protocols for gene deletions, or N- and C-terminal tagging as well as a website to design overlapping oligonucleotides for the PCRs (http://leishgedit.net/, [20]).

Casein Kinase 1 isoform 1 (CK1.1, LdBPK_351020.1) is a member of the CK1 family, which are signalling serine/threonine protein kinases involved in the regulation of various cellular processes such as the cell cycle or protein trafficking [21]. They contain a highly conserved kinase domain and a specific C-terminal domain that plays a key role in their regulation and their localization [21, 22]. In* Leishmania*, there are six isoforms, of which only two have been studied: LdCK1.4 (LdBPK_2716800.1) and LdCK1.2 (LdBPK_351030.1). LdCK1.4 is secreted by the promastigotes and may play an important role in virulence and parasite survival [23]. LdCK1.2 (LdBPK_351030.1) is an ecto-/exokinase released in the host cell via exosomes [24]. We showed that this kinase is essential for parasite survival in mammalian cells [25] and represents a validated drug target [26]. In contrast, LdCK1.1 has not yet been studied. Data available from transcriptomic analyses suggest that CK1.1 is upregulated in metacyclic promastigotes and in intracellular amastigotes [27, 28], whereas proteomic data indicates that it is a very low-abundance protein and, contrary to LdCK1.2, has not been detected in exosomes [24, 29].

In this study, we first generated a* Leishmania donovani* Bob cell line expressing Cas9 and T7 RNA polymerase. In order to validate the CRISPR Cas9 toolkit in* Leishmania donovani*, we targeted PF16 gene, which encodes a central pair protein of the axoneme, essential for parasite motility. We successfully deleted the PF16 gene, which resulted in loss of motility, and we obtained the expected flagellar localization of PF16, by C-terminal tagging. We then applied the CRISPR Cas9 toolkit for a first functional genetic analysis of CK1.1. We showed that tagged CK1.1 protein was detected in both life stages but at a very low level. We demonstrated that CK1.1 was not essential for parasite survival as the null mutant parasites could survive as promastigotes and axenic amastigotes but may have a function in stationary phase.

## 2. Material and Methods

### 2.1. *Leishmania donovani *Culture and Axenic Amastigote Differentiation

Axenic* L. donovani *strain 1S2D (MHOM/SD/62/1S-CL2D) clone LdBob was obtained from Steve Beverley, Washington University School of Medicine, St. Louis, MO, and cultured as described previously [30–32]. Briefly, 10^5^ logarithmic promastigotes per mL were incubated at 26°C in M199 media (Gibco) supplemented with 10% heat-inactivated FCS, 20 mM HEPES, pH 6.9, 4.1 mM NaHCO_3_, 2 mM glutamine, 8 *μ*M 6-biopterin, 10 *μ*g/mL folic acid, 100 *μ*M adenine, 30 *μ*M hemin, 1x RPMI 1640 vitamins solutions (Sigma), 100 U/mL of Penicillin/Streptomycin (Pen/Step), and adjusted at pH 7.4. Axenic amastigotes were obtained by incubating 10^6^ logarithmic promastigotes per mL at 37°C and 5% CO_2_ in RPMI 1640 + GlutaMAX™ -I medium (Gibco) supplemented with 20% of heat-inactivated FCS, 28 mM MES, 2 mM glutamine, 1x RPMI 1640 amino acid mix (Sigma), 1x RPMI 1640 vitamins solutions (Sigma), 10 *μ*g/mL folic acid, 2 mM glutamine, 100 *μ*M adenine, 100 U/mL of Pen/Step, and adjusted at pH 5.5. Relevant selective drugs were added to the medium at the following concentrations: 30 *μ*g/mL hygromycin B (Invitrogen), 30 *μ*g/mL puromycin dihydrochloride (Sigma), and 20 *μ*g/mL blasticidin S hydrochloride (Invitrogen). When appropriate, axenic amastigotes cell aggregates were dispersed by passing cell suspensions five times through a 27-gauge needle before analysis.

### 2.2. Analysis of the Percentage of Cell Death, Parasite Concentration, and mNeonGreen Fluorescence Intensity

Cultured parasites were diluted in DPBS (Gibco) and incubated with 2 *μ*g/mL propidium iodide (Sigma-Aldrich). Cells were analysed with a CytoFLEX flow cytometer (Beckman Coulter, Inc.) to determine the incorporation of propidium iodide (ex*λ* = 488 nm; em*λ* = 617 nm) and to monitor mNG levels in PF16::mNG::3xMyc (PF16-mNG-myc) or CK1.1::mNG::3xMyc (CK1.1-mNG-myc) transgenic parasites (ex*λ* = 506 nm; em*λ* = 517 nm). The percentage of cell death, cell growth, and the mean mNeonGreen (mNG) fluorescence intensity were calculated using CytExpert (v2.0.0.153) software (Beckman Coulter, Inc.). Graphs were generated with GraphPad Prism (v7.03).

### 2.3. Parasite Transfection

Parasite transfections were performed as described previously [20]. 1 × 10^7^ LdBob cells in logarithmic phase were transfected with 15 *μ*g of pTB007, with or without PCR reactions (mock) in 1x Tb-BSF buffer (90 mM sodium phosphate, 5 mM potassium chloride, 0.15 mM calcium chloride, 50 mM HEPES, pH 7.3) [33] using 2 mm gap cuvettes (MBP) with program X-001 of the Amaxa Nucleofector IIb (Lonza Cologne AG, Germany). Transfected cells were immediately transferred into 5 mL prewarmed medium in 25 cm^2^ flasks and left to recover overnight at 26°C before adding or not the appropriate selection drugs. Survival of drug-resistant transfectants became apparent 7–10 days after transfection.

### 2.4. PCR-Amplification of the Targeting Fragments and the sgRNA Templates

PCR reactions were performed as described previously [20]. Briefly, for the PCR-amplification of the targeting fragments of pPLOT and pT cassettes, 30 ng circular pPLOT or pT plasmid, 0.2 mM dNTPs, 2 *μ*M each of gene-specific forward and reverse primers, and 1 unit HiFi polymerase (Roche) were mixed in 1x HiFi reaction buffer (Roche), supplemented with 1.875 mM MgCl_2_ to reach a final concentration of 3.375 mM and 3% (v/v) DMSO. The PCR conditions were as follows: 5 min at 94°C then 40 cycles of 30 s at 94°C, 30 s at 65°C, and 2 min 15 s at 72°C, and lastly a final elongation step of 7 min at 72°C. The presence of the expected product was assessed by running 2 *μ*L of the 40 *μ*L reaction on a 1% agarose gel. The sample was then heat-sterilized at 94°C for 5 min and used for transfection without further purification. Primer sequences are detailed in Table S1 in Supplementary Materials.

In order to amplify the sgRNA templates, 0.2 mM dNTPs, 2 *μ*M each of primer G00 (sgRNA scaffold), 2 *μ*M of gene-specific forward primer, and 1 unit HiFi polymerase (Roche) were mixed in 1x HiFi reaction buffer with MgCl_2_ (Roche). The PCR conditions were 30 s at 98°C followed by 35 cycles of 10 s at 98°C, 30 s at 60°C, and 15 s at 72°C and a final elongation step of 7 min at 72°C. To assess the presence of the expected product, 2 *μ*L of the 20 *μ*L reaction was run on a 1% agarose gel. The sample was heat-sterilized at 94°C for 5 min and transfected without further purification. Primer sequences are detailed in Supplementary Materials in Table S1.

### 2.5. Diagnostic PCR

To assess the loss of the target gene in the knockout cell lines, genomic DNA was isolated from parasites collected after 1 passage post-transfection with the DNeasy Blood & Tissue Kit (Qiagen). One hundred nanograms of genomic DNA was mixed with 0.3 mM dNTPs, 0.5 *μ*M forward primer and reverse primer, 3% (v/v) DMSO, 2.5 units LongAmp Taq DNA polymerase (NEB), and 1x LongAmp Taq Reaction Buffer supplemented with Mg^2+^ (2 mM final, NEB). The PCR conditions were 5 min at 94°C followed by 35 cycles of 30 s at 94°C, 30 s at 60°C, 2 min 30 s at 65°C, and a final elongation step of 10 min at 72°C. Three microliters of reaction was then run on a 1% agarose gel to assess for the presence of the expected product. Primer sequences are detailed in Supplementary Materials in Table S2.

### 2.6. Protein Extraction, SDS-PAGE, and Western Blot Analysis

Between 5 × 10^7^ and 2 × 10^8^ logarithmic phase parasites (depending on the experiment) were resuspended in RIPA lysis buffer containing 150 mM NaCl, 1% Triton X-100, 20 mM Tris HCl, pH 7.4, 1% Nonidet P-40, 1 mM EDTA, and inhibitor cocktails for proteases (Roche Applied Science, IN) and supplemented with 1 mM sodium orthovanadate and 1 mM PMSF. The cells were sonicated using the Bioruptor® (Diagenode) with the high power mode for 5 min (sonication cycle: 10 sec ON, 20 sec OFF) followed by 5 more minutes (sonication cycle: 30 sec ON, 30 sec OFF) and then centrifuged. Total protein quantity was assessed by the Pierce Coomassie Plus (Bradford) Assay. Twenty micrograms of total proteins was denatured, separated by SDS-PAGE, and transferred onto polyvinylidene difluoride (PVDF) membranes (Pierce). Depending on the experiment, proteins were revealed as described in Supplementary Materials in Table S3, using the following primary antibodies at the indicated dilutions: (i) anti-CK1.2 antibody (1/500, [25]), (ii) anti-myc antibody (1/1000, Biosensis R-1319-100), anti-Flag M2 antibody (1/1000, Sigma F3165); and secondary antibodies: anti-rabbit antibody (1/20000, Thermo Scientific 31462) and anti-mouse antibody (1/20000, Thermo Scientific 32230). Proteins were revealed by SuperSignal™ West Pico Chemiluminescent Substrate (Thermo Scientific) using the PXi image analysis system (Syngene) at various exposure times.

### 2.7. Fluorescence Microscopy

*L. donovani* promastigotes expressing the fluorescent fusion protein PF16-mNG-myc were imaged by live microscopy. Samples were prepared as previously described [34]. Briefly, parasites were harvested from logarithmic phase culture by centrifugation at 800*g* for 5 min and washed three times in PBS with Hoechst 33342 at 5 *μ*g/mL. The cells were resuspended in 50 *μ*L PBS, and 2 *μ*L was placed on a microscope slide, then a coverslip was applied, and the cells were immediately imaged with a 60x NA 1.42 plan-apochromat oil immersion objective lens (Olympus AMEP4694) on a EVOS FL microscope (Thermo Fischer Scientific, AMF4300) with a ICX445 monochrome charge-coupled device (CCD) camera (Sony) at room temperature.

### 2.8. Parasite Tracking

*Leishmania *promastigotes from early stationary phase were filmed for 10 s (200 frames) with a Leica DMI 4000B microscope, using a 40x objective and an Evolv EMCCD camera, with Metaview software. Tracking was performed using the Spot Detector and Spot Tracking tools from Icy software [2], with defaults settings and the following modifications: Spot Detector: Scale 3, Sensitivity 80 (~7 pix); size filtering: min = 10 – max = 300.

### 2.9. Bioinformatics

Multiple sequence alignments (MSA) were computed using the PSI-Coffee mode of T-Coffee [35]. The resulting alignments were visualized using Clustalw (1.83).

## 3. Results and Discussion

### 3.1. Expression of Cas9 and T7 RNA Polymerase Does Not Affect Parasite Growth and Differentiation

**Figure 1:**
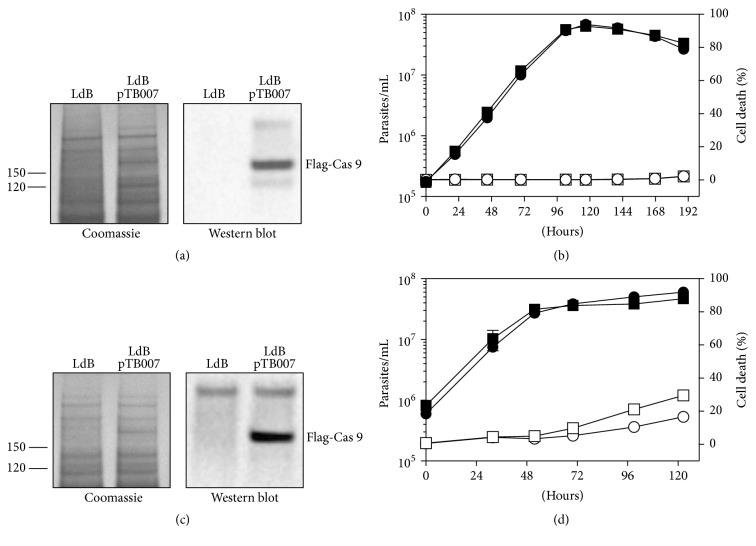
*Constitutive expression of Cas9 in L. donovani Bob strain*. (a) Proteins were extracted from LdBob (LdB) or LdBob expressing Cas9-FLAG (LdB pTB007, 162 kDa) promastigote in logarithmic phase. Twenty micrograms was analysed by Western blotting using the anti-FLAG M2 antibody (right panel). The Coomassie-stained membrane of the blot is included as a loading control (left panel). Protein weight in kDa is indicated on the left. (b) Logarithmic phase promastigotes were seeded at 1 × 10^5^ promastigotes/mL and cultured for 8 days. Samples were collected every 24 h to assess cell number (black symbol) and percentage of cell death (white symbol) by flow cytometry in triplicate in two independent experiments. Cell lines: LdB (square) and LdB pTB007 (circle). (c) Proteins were extracted from LdBob or LdBob expressing Cas9-FLAG (LdB pTB007, 162 kDa) axenic amastigotes (48 h after temperature and pH shift) and processed as described in (a). (d) Logarithmic phase promastigotes were seeded at 1 × 10^6^ promastigotes/mL, shifted to 37°C and pH 5.5 and cultured for 5 days. Samples were collected and treated as described in (c).

We first transfected LdBob promastigotes with the plasmid pTB007 (LdB pTB007) expressing (i) the humanized* Streptococcus pyogenes *Cas9 nuclease gene* (hSpCas9)* [36] with a nuclear localization signal and three copies of the FLAG epitope at the N-terminus, (ii) T7 RNA polymerase (T7 RNAP) and (iii), a hygromycin resistance gene [20]. We confirmed the expression of Cas9 by Western blot analysis (Figure 1(a)) and showed that this expression did not alter the growth of* L. donovani* promastigotes (Figure 1(b)), similar to what has been shown with* L. mexicana* promastigotes [20]. We found that Cas9 is also expressed in axenic amastigotes (Figure 1(c)). The presence of pTB007 did not interfere with axenic amastigote differentiation or proliferation (Figure 1(d)) but surprisingly has a slight positive effect on cell survival in late stationary phase.

### 3.2. Validation of the CRISPR Cas9 Gene Editing Toolkit in* L. donovani*

To assess the efficiency of the CRISPR Cas9 gene editing toolkit in* L. donovani*, we performed C-terminal tagging and the generation of null mutants on the well-studied* PF16* gene (LdBPK_201450.1), which encodes a central pair protein of the flagellar axoneme. Previous experiments in* L. mexicana* showed flagellar localization of PF16, and its deletion abrogates parasite motility [20]. We sought to replicate these phenotypes in* L. donovani* using the same gene editing strategy to validate the toolkit in this parasite species.

To fuse PF16 with the mNeonGreen-3xmyc (mNG-myc) in LdBob, we produced two PCR fragments: the donor DNA cassette, containing mNG-myc and the puromycin-resistance marker, as well as the sgRNA template to generate a Cas9 cleavage downstream of the* PF16 *gene [20]. It is crucial to know the exact sgRNA sequence and protospacer-adjacent motif (PAM) to successfully tag or delete genes in* Leishmania* spp. using CRISPR Cas9. There are important differences between the genome of LdBPK282A1 reference strains from South-Eastern Nepal [37] and that of LdBob, a strain derived from the Sudanese isolate Ld1S2D (MHOM/SD/62/1S-CL2D [38]). Thus, we used an unpublished Ld1S2D reference genome (PRJNA396645, https://www.ncbi.nlm.nih.gov/bioproject/396645) to design the primers required to generate the donor DNA and the corresponding sgRNAs. Sequences were identified using the EuPaGDT CRISPR gRNA Design Tool [39] with similar target parameters as those used in the LeishGEdit strategy [20]. LdB pTB007 promastigotes were then transfected with the two PCR fragments. The transgenic parasites were selected using puromycin; the correct integration of the tagging cassette was confirmed by the detection of the tagged protein using microscopy and Western blot analysis with an anti-myc antibody (Figures S1A and B). The localization of the tagged protein was consistent with the known localization of PF16 [20]. Fluorescence intensity measurements showed that its abundance is constant during promastigote growth and that the expression of the mNG-myc reporter fused to the C-terminus of PF16 does not lead to any growth defects (Figure S1C). These data indicate that the tagging of PF16 using CRISPR Cas9 was successful in* L. donovani*. This approach is simple and fast and will greatly improve the way we study* Leishmania* genes compared to previous methods of gene tagging (e.g., by expressing GOI fused to a fluorescent tag from an episome), since part of their endogenous regulation may be better maintained through the conservation of either the 5′ or 3′UTR (depending whether the tagging is at the C- or N-terminus, resp.).

**Figure 2:**
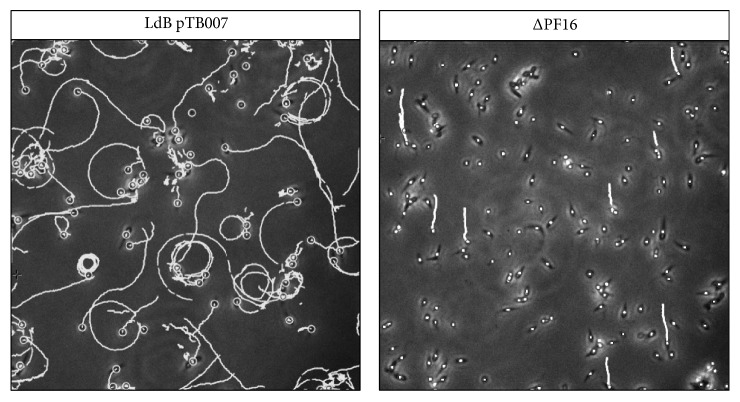
*L. donovani PF16 null mutants are immotile.* LdB pTB007 or LdB pTB007* ΔPF16* promastigotes in logarithmic phase were filmed for 10 s (200 frames). The tracking of individual parasites was performed using the Spot Detector and Spot Tracking tools from Icy software. Images were taken with a Leica DMI 4000B microscope, using a 40x objective and an Evolv EMCCD camera, with Metaview software.

Next, we targeted the* PF16* locus to generate null mutants in a single round of transfection. LdB pTB007 promastigotes were transfected with four PCR fragments corresponding to the two sgRNA templates to generate a double-strand break upstream and downstream of the* PF16* CDS, and the two repair cassettes containing the resistance marker genes for blasticidin and puromycin [20]. We confirmed the generation of a double drug-resistant cell population by PCR (Figure S2A), indicating that* PF16* has been successfully deleted in the whole population, without the need for subcloning as observed previously with* L. mexicana *and* L. major* [20]. The ΔPF16 mutant grew similarly to the parental strain (data not shown), only displaying a loss of motility (Figure 2), which is consistent with published data [20, 40, 41]. Altogether, these data validate the use of the CRISPR Cas9 toolkit developed by Beneke et al. in* L. donovani *[20]. This approach will overcome the two main limitations for the genetic manipulation of* L. donovani*. First, because* L. donovani* adapts very fast to its environment by copy number variations [15], the ability to generate homozygote knockouts in one single transfection will minimise the introduction of compensatory mutations, which could mask the phenotype of the knockouts. This is therefore a major improvement compared to previous methods for gene deletion [42]. Second,* L. donovani *strain Ld1S2D, purified from the spleen or the liver of infected hamsters, is particularly sensitive to* in vitro* culture, as this strain loses virulence after only 5 to 10 passages in culture to become unable to infect hamsters ([38] and our unpublished data). Thus, minimising time in culture is a prerequisite for virulence studies; hence this CRISPR Cas9 method will enhance our ability to conduct such studies in* L. donovani*, one of the causative agents of the only lethal form of leishmaniasis.

### 3.3. *Leishmania* CK1.1 Member of the Casein Kinase Family Is More Closely Related to CK1.2 Than to Other CK1s

**Figure 3:**
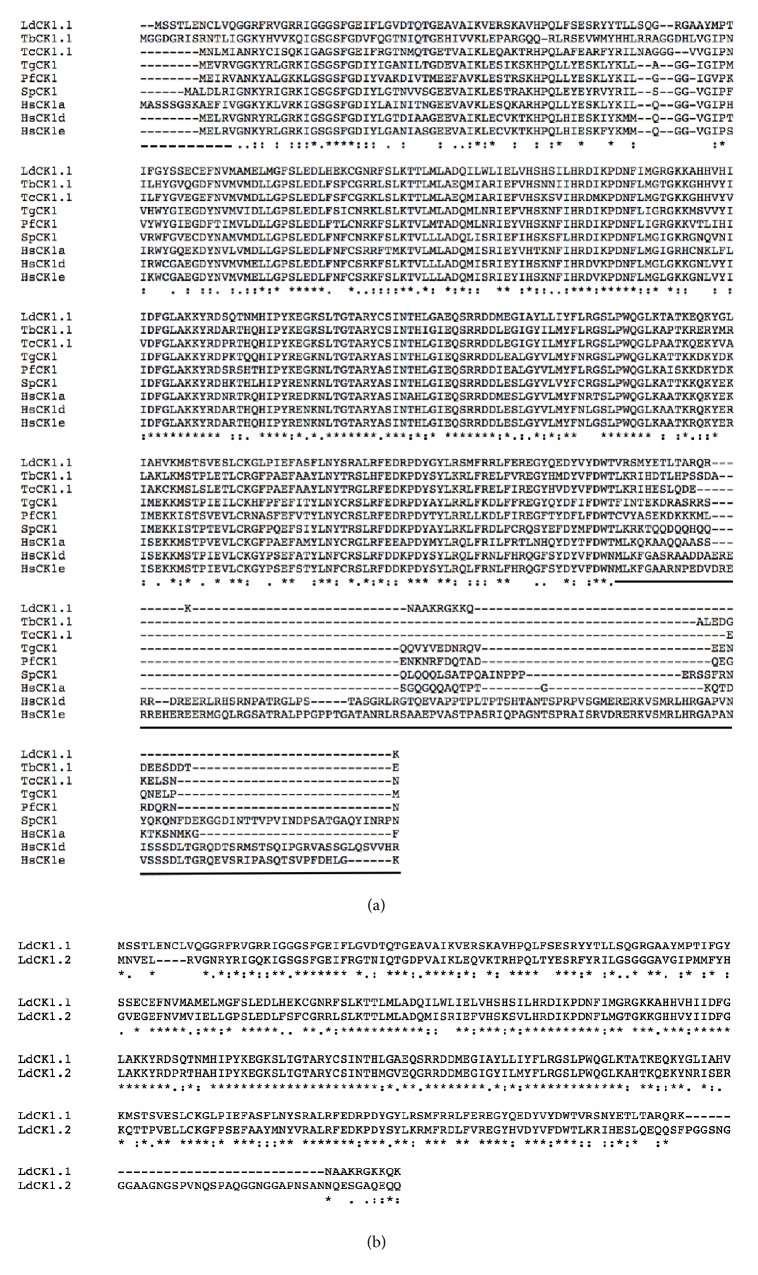
*Amino acid sequence alignment of Leishmania Casein Kinase I proteins*. (a) The amino acid sequences of LdCK1.1 (*Leishmania donovani* LdBPK_351020.1, E9BRX8), TbCK1.1 (*Trypanosoma brucei* Tb927.5.790, Q57W24), TcCK1.1 (*Trypanosoma cruzi* TcCLB.508541.220, Q4DN97), TgCK1 (*Toxoplasma gondii* CK1, Q6QNM1), PfCK1 (*Plasmodium falciparum* CK1, C6S3F7), SpCK1 (*Schizosaccharomyces pombe* hhp1, P40235), hsCK1*α* (*human* CSNK1A1, P48729), HsCK1*δ* (*human* CSNK1D, P48730), and HsCK1*ε* (*human* CSNK1E, P49674) have been compared and the alignments were computed using the M-Coffee mode of T-Coffee. The resulting alignments were visualized using Clustalw. *∗* corresponds to amino acid residues that are invariant in all four CK1s. The dotted line marks the N-terminal domain, the black line marks the C-terminal domain, and the rest is the kinase domain. (b) The amino acid sequences of LdCK1.1 and LdCK1.2 (LdBPK_351030.1) have been compared and the alignments were computed using the M-Coffee mode of T-Coffee. The resulting alignments were visualized using Clustalw. *∗* corresponds to amino acid residues that are invariant in all four CK1s.

LdCK1.1 (LdBPK_351020.1) is 324 amino acids long and has a predicted molecular weight of 37.2 kDa. It contains a kinase domain, a N-terminal domain longer than that of CK1 of other eukaryotes including LdCK1.2, but similar to that of TbCK1.1, and a C-terminal domain shorter than that of the human CK1 (*α*, *δ*, and *ε*), LdCK1.2, or TbCK1.2, but similar to that of TbCK1.1 (Figure 3(a)). LdCK1.1 is closely related to LdCK1.2, with 67% identity in protein sequence [25]. As the two encoding genes are adjacent on chromosome 35, they probably originated from the same gene that duplicated and then evolved differently [25]. The most striking difference is the lack of 33 amino acids in the C-terminal domain of LdCK1.1 compared to that of LdCK1.2 (Figure 3(b)). Since the C-terminal domain is particularly important for the localization and the regulation of CK1 family members, these data suggest that CK1.1 and CK1.2 could have different localization and function [22]. LdCK1.1 has an orthologue in* T. brucei* TbCK1.1 (Tb927.5.790, 60% identity) and in* T. cruzi* TcCK1.1 (TcCLB.508541.220, 63% identity), which are also adjacent to TbCK1.2 and TcCK1.2, respectively. The two isoforms present in* T. cruzi* have a distinct feature compared to the orthologues in other trypanosomatids; TcCK1.2 is encoded by an array of five copies, while TcCK1.1 is encoded by a single copy (CL Brener-Esmeraldo-like strain [43]). Finally, LdCK1.1 shows 57% identity with the human CK1 *δ*, compared to 67% for LdCK1.2. Previously we showed that* Leishmania* CK1.2 is the most conserved kinase in* Leishmania*, and the kinase with the most similarity to its human orthologues [25], leading to the hypothesis that CK1.2 could have a function outside of the parasite by mimicking the host CK1. These characteristics are not shared by CK1.1, suggesting that it could be essentially intracellular; this hypothesis is supported by proteomics data showing that CK1.1 is not detected in* Leishmania* exosomes [24].

### 3.4. *Leishmania donovani* CK1.1 Is a Nonessential Kinase Which May Play a Role in Stationary Phase

Next, we applied the CRISPR Cas9 toolkit to gain insight into CK1.1 function in the parasite. We tagged the protein to determine its localization and we deleted it to determine whether this kinase is essential for promastigote or amastigote survival.

**Figure 4:**
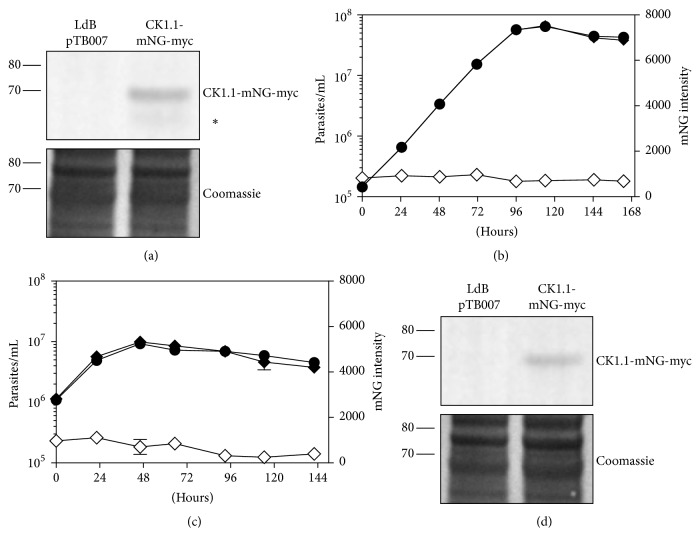
*Successful in locus tagging of L. donovani CK1.1 with mNG-myc tag.* (a) Proteins were extracted from LdB pTB007 or LdB CK1.1-mNG-myc promastigotes in logarithmic phase and twenty micrograms was analysed by Western blotting using an anti-Myc tag antibody (Top panel). The Coomassie-stained membrane of the blot is included as a loading control (bottom panel). Protein weight in kDa is indicated on the left. The expected size of the fusion protein is 69,8 kDa. The lower band indicated with an asterisk (*∗*) may be a result of protein degradation. (b) Promastigotes were seeded at 1 × 10^5^ promastigotes/mL and cultured for 7 days, and aliquots were taken every 24 h for analysis. Cell number (black symbol) and mNeonGreen fluorescence intensity (white symbol) were assessed by flow cytometry in triplicate in two independent experiments. Fluorescence intensity of the LdB pTB007 strain was used for normalization. Cell lines: LdB pTB007 (circle) and LdB pTB007 CK1.1-mNG-myc (diamond). (c) Similar to (b), except that promastigotes were seeded at 1 × 10^6^ promastigotes/mL, shifted to 37°C and pH5.5 and cultured for 6 days. (d) Similar to (a), except that proteins were extracted from LdB pTB007 or LdB CK1.1-mNG-myc axenic amastigotes (48 h after temperature and pH shift).

To generate transgenic parasites expressing CK1.1-mNG-myc from the endogenous locus, we cotransfected LdB pTB007 promastigotes with a sgRNA cassette targeting the 3′ end of* CK1.1* and a repair cassette containing the puromycin-resistance marker and the mNG-myc tag in frame. We then performed a Western blot analysis to determine whether CK1.1-mNG-myc was expressed in the transfected promastigotes and revealed a band at about 70 kDa corresponding to the expected size of the tagged protein (Figure 4(a)). Using FACS analysis to measure cell density, we showed that the parasites expressing CK1.1-mNG-myc displayed no change in growth phenotype at the promastigote stage (Figure 4(b)). CK1.1 shows comparable expression in logarithmic and stationary phase (Figure 4(b)), although at a lower level than PF16-mNG-myc, as judged by Western blot and FACS analysis (Figures S1B and S1C). Promastigotes expressing CK1.1-mNG-myc could differentiate into axenic amastigotes that proliferated at a rate similar to the control cells (Figure 4(c)). CK1.1-mNG-myc is also detected in amastigotes, as shown Figure 4(d), with slightly higher levels in logarithmic phase than in stationary phase (Figure 4(c)). Altogether, these data suggest that CK1.1 is a low-abundance protein, explaining why we could barely detect the protein above background, using a fluorescence microscope (data not shown). This finding is consistent with proteomic data showing that CK1.1 in* L. donovani *or in* L. major *[30] could only be detected with 1 peptide [29] and in* T. brucei* where TbCK1.1 was not detected contrary to TbCK1.2 [44, 45].

**Figure 5:**
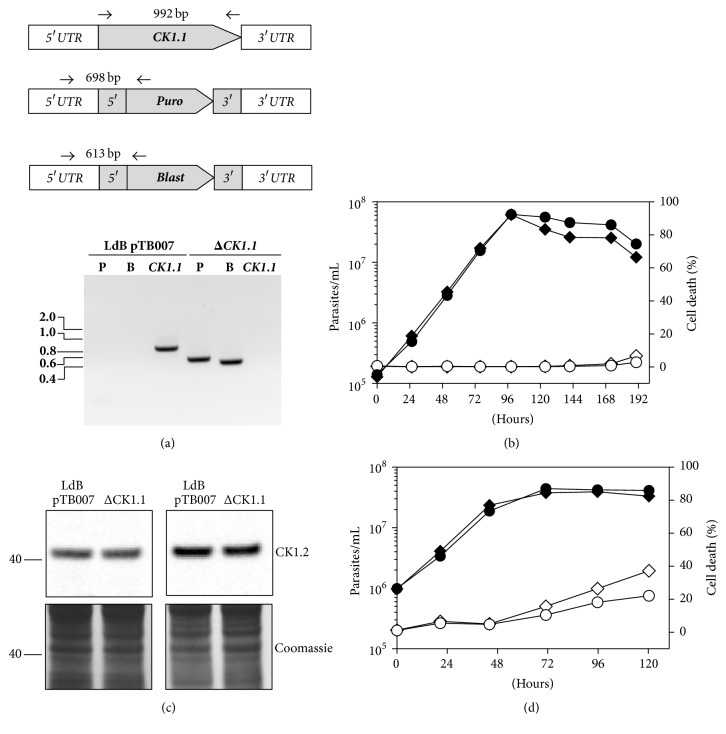
*CK1.1 is nonessential in L. donovani*. (a) PCR analysis of the* ΔCK1.1* cell line. Diagrams showing the* CK1.1* locus and PCR primers (small arrows) used to test for the presence of the* CK1.1* CDS or the correct integration of puromycin and blasticidin drug-resistance genes (top panel). PCR products run on an agarose gel to assess the correct integration of the puromycin-resistance gene (P), blasticidin-resistance gene (B), and the presence/absence of the* CK1.1* CDS (bottom panel). Fragments sizes in kb are indicated on the left. (b) Promastigotes were seeded at 1 × 10^5^ promastigotes/mL and were cultured for 8 days. Aliquots were taken every 24 h to assess cell number (black symbol) and percentage of death (white symbol) by flow cytometry in triplicate from two independent experiments. Cell lines: LdB pTB007 (circle), LdB pTB007 ΔCK1.1 (diamond). (c) Proteins were extracted from LdB pTB007 or LdB pTB007 ΔCK1.1 promastigotes in logarithmic phase (right panel) and axenic amastigotes 48 h after temperature and pH shift (left panel) and twenty micrograms was analysed by Western blotting using an anti-CK1.2 antibody (Top panel). The Coomassie-stained membrane of the blot is included as a loading control (bottom panel). Protein weight is in kDa is indicated on the left. (d) Similar to (b), except that promastigotes were seeded at 1 × 10^6^ promastigotes/mL, shifted to 37°C and pH 5.5 and cultured for 6 days.

To determine whether CK1.1 is essential for parasite survival, we generated a CK1.1 null mutant in* L. donovani*. We performed a cotransfection of LdB pTB007 promastigotes with two sgRNA cassettes targeting the 5′ and the 3′ end of* CK1.1* and two repair cassettes containing, respectively, the puromycin and the blasticidin-resistance genes. We obtained parasites that were resistant to both drugs and confirmed the correct integration of the puromycin and blasticidin-resistance genes at* CK1.1* locus and the complete loss of* CK1.1* by PCR as shown in Figure 5(a). Similarly to* PF16*,* CK1.1* was deleted in the whole population in one single transfection, confirming once again the remarkable efficiency of this method. The generation of homozygous ΔCK1.1 parasites indicates that LdCK1.1 is not essential for promastigote survival. We did not observe any growth defect except in stationary phase, where the cell density of the ΔCK1.1 was lower than that of the control parasites (Figure 5(b)). Although the percentage of cell death, as measured by propidium iodide (PI) incorporation, was slightly higher in ΔCK1.1 parasites (4%) than in control parasites (2%), it remained below 5% which thus could not entirely explain the decrease in cell density. However, when cells contain fragmented DNA or when they lose their DNA (zoids), the percentage of PI^+^ cells no longer corresponds to the real percentage of dead cells. We investigated this hypothesis by measuring the ΔCK1.1 DNA content in stationary phase using FACS analysis, and found no differences in the DNA content of ΔCK1.1 compared to that of control parasites (data not shown). We did not observe an increase in fragmented DNA or cell debris (data not shown), suggesting that the difference in cell density might not be a consequence of cell death. Interestingly, these data indicate that CK1.2 cannot compensate for the loss of CK1.1 and conversely, the absence of CK1.1 does not influence the regulation of CK1.2 abundance. Indeed, there is no difference in CK1.2 level between the mutant and the control strains in promastigotes (Figure 5(c), left panel) or in amastigotes (Figure 5(c), right panel). These results suggest the absence of a regulatory feedback loop between the two kinases, supporting the hypothesis that they might have different functions. We did not observe any morphological differences between ΔCK1.1 and control parasites (data not shown).

We investigated whether promastigotes could undergo axenic amastigote differentiation in the absence of CK1.1. We showed that they could differentiate and similar to what we observed in promastigotes, they could proliferate as well as control parasites (Figure 5(d)); however, ΔCK1.1 parasites present a higher percentage of cell death in stationary phase (about 40%) than the control parasites (about 20%). This cell mortality, exclusively restricted to the amastigote stage, as we did not observe this phenomenon in promastigotes (Figure 5(b)), indicates that CK1.1 could have a role in late stationary phase. These data demonstrate that CK1.1 is not essential for amastigote survival, which is consistent with observations in* T. brucei* showing that TbCK1.1 is not essential for bloodstream form survival contrary to TbCK1.2 [46].

Overall, our data demonstrate that CK1.1 is not essential for parasite survival and axenic amastigote differentiation but could have a role in the regulation of processes linked to stationary growth phase. Conversely, we have previously shown that CK1.2 is essential for the survival of axenic and intracellular amastigotes [25], suggesting these two related kinases have evolved independently. Evolution of the two isoforms is similar in* T. brucei* where TbCK1.2 is essential for cell survival, but not TbCK1.1 [46]. Altogether, these data suggest that CK1.1 and CK1.2 have evolved similarly in the two major trypanosomatids.

## 4. Conclusion

In this study, we validated the CRISPR Cas9 toolkit for* Leishmania donovani* targeting PF16. Gene editing and particularly PCR-based CRISPR Cas9 methods will have a major impact on our ability to study the biology of* L. donovani*. The fact that only one single transfection is required to obtain knockout mutants will (i) dramatically limit parasite adaptation, by decreasing any compensatory mutation that could mask the phenotype, and (ii) allow the use of hamster-derived parasites for genetic manipulation by preventing the nonspecific loss of virulence occurring during* in vitro* culture. The use of CRISPR Cas9 in hamster-derived* L. donovani* opens new possibilities of studying the phenotype of nonessential genes in the context of the relevant mammalian host, thus moving beyond* in vitro* studies for the medically most relevant* Leishmania* spp.

## Supplementary Materials

Supplementary Materials Figure S1. Characterisation of PF16-mNG-myc. Figure S2. Generating PF16 null mutant in* L. donovani*. Table S1. Primers used to tag and knockout PF16 and CK1.1 in* L. donovani* using pT/pPLOT plasmids. Table S2. Primers used for the validation of PF16 and CK1.1 Knockouts. Table S3. Conditions for Western blot analysis.
