# Supplementary material for: Characterisation of Casein Kinase 1.1 in Leishmania donovani Using the CRISPR Cas9 Toolkit
Source: Biomed Res Int. 2017 Nov 29;2017:4635605. doi: 10.1155/2017/4635605 (PMC5733176; doi:10.1155/2017/4635605)
Supplement: Supplementary Materials — Figure S1. Characterisation of PF16-mNG-myc. Figure S2. Generating PF16 null mutant in L. donovani. Table S1. Primers used to tag and knockout PF16 and CK1.1 in L. donovani using pT/pPLOT plasmids. Table S2. Primers used for the validation of PF16 and CK1.1 Knockouts. Table S3. Conditions for Western blot analysis. [file 4635605.f1.pdf]

Figure S1

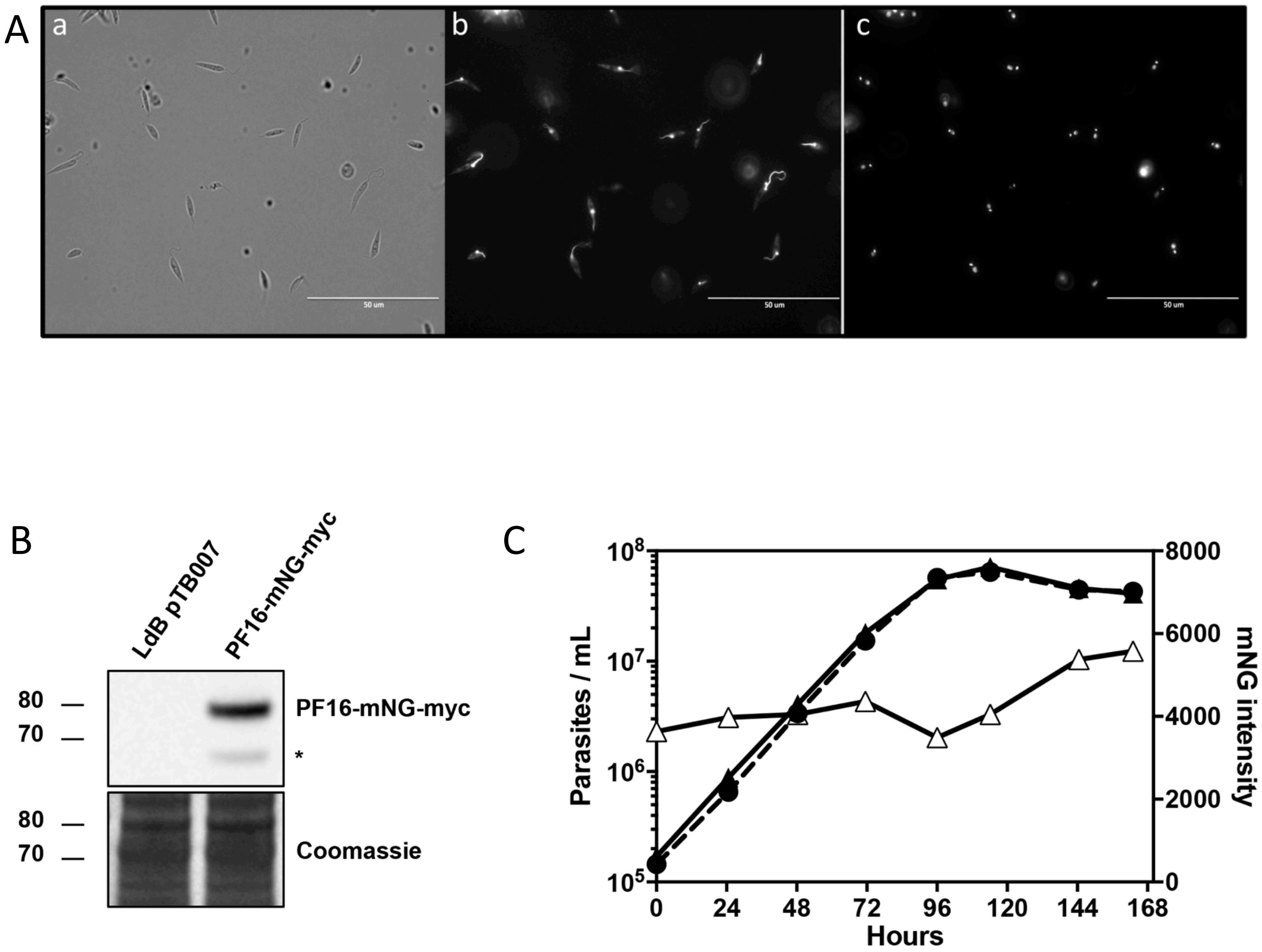

<u>FIGURE S1. Characterisation of PF16-mNG-myc.</u>

(A) Fluorescence micrographs of *Ld*B pTB007 promastigotes expressing PF16-mNG-myc. 100% parasites showed a flagellar localisation in passage 1 after transfection. (a) Transmitted light, (b) mNeonGreen fluorescence, (c) Hoechst-stained DNA fluorescence. **(B)** Proteins were extracted from LdB pTB007 or LdB pTB007 PF16-mNG-myc promastigotes in logarithmic phase and twenty micrograms were analysed by Western blotting using an anti-Myc antibody (Top panel). The Coomassie-stained membrane of the blot is included as a loading control (Bottom panel). The lower band indicated with an asterisk (*) may be a result of protein degradation. Protein weight in kDa is indicated on the left. **(C)** Promastigotes were seeded at 1 x 10^5 promastigotes/ml and were cultured for 7 days. Aliquots were taken every 24h to assess cell number (black symbol) and mNeonGreen fluorescence intensity (white symbol) by flow cytometry in triplicates from two independent experiments. Cell lines: LdB pTB007 (circle), LdB pTB007 PF16-mNG-myc (triangle). The dotted line denotes data that are identical to those used in Figure 4B.

Figure S2

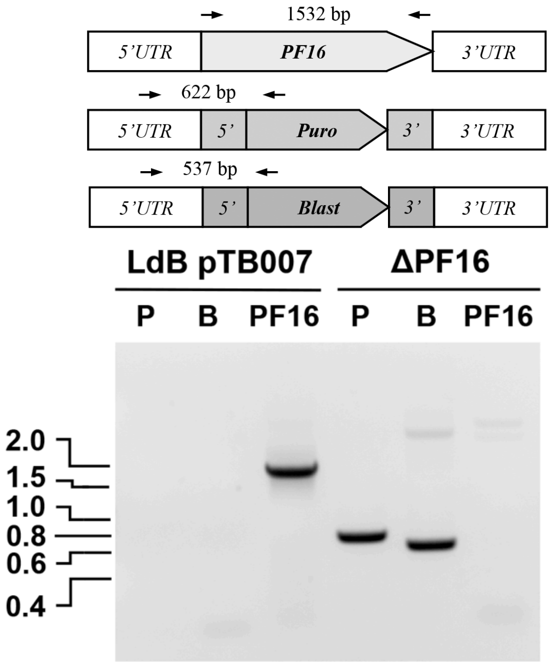

FIGURE S2. Generating PF16 null mutant in L. donovani

PCR analysis of the ΔPF16 cell line. Diagrams showing the PF16 locus and PCR primers (small arrows) used to test for the presence of the PF16 CDS or the correct integration of puromycin and blasticidin drug-resistance genes (Top panel). PCR products run on an agarose gel to assess the correct integration of the puromycin-resistance gene (P), blasticidin-resistance gene (B) and the presence/absence of the PF16 CDS (Bottom panel). Fragments sizes in kb are indicated on the left.

Table S1: Primers used to tag and knockout PF16 and CK1.1 in *L. donovani* using pT/pPLOT plasmids

L. donovani PF16 5'HF forward	5' ATCGCTGCAGAAGTGATTCTCCCCGTCCCCgtataatgcagacctgctgc 3'
L. donovani PF16 3'HF forward	5' AAGATCGAGAACTACCACGTGCAGCAGCACggttctggtagtggttccgg 3'
L. donovani PF16 3'HF reverse	5' CGAGCAGCGTGAGTTGGCGTGGCCGTGCCGccaatttgagagacctgtgc 3'
L. donovani PF16 5'sgRNA forward	5' gaaattaatacgactcactataggTGTGGGCACGGCTATTGAGTgttttagagctagaaatagc 3'
L. donovani PF16 3'sgRNA forward	5' gaaattaatacgactcactataggGCTGATGCTCAGCCGCCATTgttttagagctagaaatagc 3'
L. donovani CK1.1 5'HF forward	5' CGCCTCTTTCCTGAACCCTGCCGTCAGCCAgtataatgcagacctgctgc 3'
L. donovani CK1.1 3'HF forward	5' AATGCAGCGAAGCGCGGAAAGAAACAGAAGggttctggtagtggttccgg 3'
L. donovani CK1.1 3'HF reverse	5' ACTTCGCCTATTCACTAACAAGCCTATCCAccaatttgagagacctgtgc 3'
L. donovani CK1.1 5'sgRNA forward	5' gaaattaatacgactcactataggGCTACTTCTCTCTTGTGTTCgttttagagctagaaatagc 3'
L. donovani CK1.1 3'sgRNA forward	5' gaaattaatacgactcactataggTGTATGTCTGTGTTCGTTAAgttttagagctagaaatagc

Underlined sequences indicate sgRNA target sites or homology regions in the genome

Table S2 Primers used for the validation of PF16 and CK1.1 Knockouts

ORF amplification	*L. donovani* PF16 ORF forward	5’ cttgctgtgccttgccaccaATGTCGAATCGGGTTATTCTGC 3’
	L. donovani PF16 ORF reverse	5’ tcccggggatatcatcgattccGTGCTGCTGCACGTGGTAG 3’
	L. donovani CK1.1 ORF forward	5’ cttgctgtgccttgccaccaATGGAGTGGAAGAGCAAG 3’
	L. donovani CK1.1 ORF reverse	5’ tcccggggatatcatcgattccCTTCTGTTTCTTTCCGCG 3’
Amplification of sequence across integration junction, *L. donovani* PF16	*L. donovani* PF16 5’UTR forward	5’ TGACATCGCTGCAGAAGTGA 3’
	Puromycin reverse	5’ TCAATGTGTCGATCTGGGTCAAC 3’
	Blasticidin reverse	5’ CCGTTGCTCTTTCAATGAGGGTG 3’
Amplification of sequence across integration junction, *L. donovani* CK1.1	*L. donovani* CK1.1 5’UTR forward	5’ TGCTCAACGACTCTCCGACGT 3’
	Puromycin reverse	5’ TCAATGTGTCGATCTGGGTCAAC 3’
	Blasticidin reverse	5’ CCGTTGCTCTTTCAATGAGGGTG 3’

Table S3 : Conditions for Western blot analysis

*PBST: PBS with 0.25% Tween 20
**TBST: Tris-buffered saline with 0.075% Tween 20

Western blot description	Blocking buffer	1st antibody dilution	Washing buffer	2nd antibody dilution
Anti-myc Tag	PBST* + 5% BSA (Sigma)<br><br>1h @RT	Anti-myc Tag (Biosensis R-1319-100)<br><br>1:1000 in PBST + 2.5% BSA (Sigma)<br><br>O/N @4°C	PBST<br><br>3 x 5 min	Anti-rabbit (Thermo Scientific 31462)<br><br>1:20000 in PBST + 2,5% BSA (Sigma)<br><br>1h @RT
Anti-FLAG M2	TBST** + 5% Milk (Lactel)<br><br>1h @RT	Anti-FLAG M2 (Sigma F3165)<br><br>1:1000 in TBST + 5% Milk (Lactel)<br><br>O/N @4°C	TBST + 5% Milk (Lactel)<br><br>3 x 5 min	Anti-mouse (Thermo Scientific 32230)<br><br>1:20000 in TBST + 5% Milk (Lactel)<br><br>1h @RT
Anti-CK1.2	PBST + 5% BSA (Sigma)<br><br>1h @RT	Anti-CK1.2 (SY3535)<br><br>1:500 in PBST + 2,5% BSA (Sigma)<br><br>O/N @4°C	PBST<br><br>3 x 5 min	Anti-rabbit (Thermo Scientific 31462)<br><br>1:20000 in PBST + 2,5% BSA (Sigma)<br><br>1h @RT
